# Vitamin D-Binding Protein in Pregnancy and Reproductive Health

**DOI:** 10.3390/nu12051489

**Published:** 2020-05-20

**Authors:** Melinda Fernando, Stacey J. Ellery, Clara Marquina, Siew Lim, Negar Naderpoor, Aya Mousa

**Affiliations:** 1 Monash Centre for Health Research and Implementation (MCHRI) and Centre of Cardiovascular Research and Education in Therapeutics (CCRET), School of Public Health and Preventive Medicine, Monash University, Melbourne 3168 VIC, Australia; melinda.fernando@monash.edu (M.F.); clara.marquina@monash.edu (C.M.); siew.lim@monash.edu (S.L.); negar.naderpoor@monash.edu (N.N.); 2 The Ritchie Centre, Hudson Institute of Medical Research and Department of Obstetrics and Gynaecology, Monash University, Melbourne 3168 VIC, Australia; stacey.ellery@hudson.org.au

**Keywords:** vitamin D-binding protein, pregnancy, reproductive health, fertility, vitamin D metabolites

## Abstract

Vitamin D-binding protein (VDBP), the main carrier of vitamin D, has recently been implicated in reproductive health and pregnancy outcomes including endometriosis, polycystic ovary syndrome (PCOS), pre-eclampsia, and gestational diabetes mellitus (GDM). Improved methods for measuring VDBP and an increased understanding of its role in biological processes have led to a number of newly published studies exploring VDBP in the context of pregnancy. Here, we synthesize the available evidence regarding the role of VDBP in reproductive health and pregnancy, and we highlight areas requiring further study. Overall, low levels of maternal serum VDBP concentrations have been associated with infertility, endometriosis, PCOS and spontaneous miscarriage, as well as adverse pregnancy outcomes including GDM, pre-eclampsia, preterm birth and fetal growth restriction. However, increased VDBP concentration in cervicovaginal fluid has been linked to unexplained recurrent pregnancy loss and premature rupture of membranes. Some genetic variants of VDBP have also been associated with these adverse outcomes. Further studies using more accurate VDBP assays and accounting for ethnic variation and potential confounders are needed to clarify whether VDBP is associated with reproductive health and pregnancy outcomes, and the mechanisms underlying these relationships.

## 1. Introduction

Pregnancy induces a natural physiological challenge to the mother, with many systems and functions of the human body adapting to this unique but ultimately temporary environment. One such system is the regulation of vitamin D and its metabolites. The main function of vitamin D is the control of calcium absorption in the small intestine, working alongside the parathyroid hormone (PTH) to mediate skeletal bone mineralization and maintain calcium homeostasis in the bloodstream [1]. Regulation of the vitamin D system is also important for extra-skeletal functions including the enhancement of muscle cell contractility, immunity, cognitive capacity and cardiometabolic health [2,3,4,5,6]. Immunomodulatory and anti-inflammatory properties of vitamin D have been demonstrated, particularly in conditions of chronic low-grade inflammation such as type 2 diabetes [7] and heart failure [8], as well as in autoimmune diseases including asthma [9]. Vitamin D requirements are increased during pregnancy to adapt to heightened physiological demands in the mother, including driving the formation of the fetal skeleton and maintaining an environment of tolerance to paternal and fetal tissue and their accompanying alloantigens [10,11].

Deficiency in vitamin D during the physiologically and metabolically challenging period of pregnancy has been associated with several adverse pregnancy and childhood outcomes including gestational diabetes, pre-eclampsia, preterm birth, childhood asthma and impaired psychomotor and cognitive development [12,13,14]. However, many of these associations have been inconsistently reported in the literature, despite extensive research into vitamin D in the context of pregnancy. Furthermore, the lack of clarity regarding optimal vitamin D levels in relation to pregnancy outcomes has led to inconsistencies in the guidelines for classifying vitamin D deficiency or defining the level of supplementation required to support a healthy pregnancy [15].

Most of the previous literature examining vitamin D in pregnancy has been limited to a single measure of vitamin D, that is, total 25-hydroxyvitamin D (25(OH)D). However, attempts to understand the complexities of the vitamin D metabolic system and its effects in pregnancy have led to the exploration of other metabolites within the vitamin D system. More novel components of the vitamin D system, such as the vitamin D-binding protein (VDBP), the primary carrier protein of vitamin D, have recently been identified as potential targets for research. This is due to an increased understanding of the potential role of VDBP in the physiology underlying vitamin D deficiency and the metabolic changes in pregnancy [16]. VDBP concentrations have been shown to increase dramatically in pregnancy and thus influence the biologically and functionally active portion of vitamin D, free vitamin D, which is postulated to be more representative of vitamin D status in the pregnancy state than total 25(OH)D. VDBP itself has been implicated as a potential biomarker of pregnancy outcomes as it has been linked to several adverse outcomes including gestational diabetes, pre-eclampsia and preterm labour, although the present literature exploring this protein remains very limited [17,18,19]. Importantly, VDBP has been linked to various biological processes that are often exacerbated or heightened in the pregnant state, including immunoregulation, glucose metabolism, and regulation of blood pressure [20]. VDBP is also involved in maintaining an environment of tolerance to paternal and fetal tissue and the augmentation of pro-inflammatory states [16].

These largely unexplored components of the vitamin D metabolic system provide alternative and novel avenues for improving our understanding of the functions of vitamin D in pregnancy, and potentially optimising pregnancy outcomes. To date, vitamin D has been studied extensively in the context of pregnancy and other health related outcomes, but VDBP has not received nearly as much attention, with a paucity of in vitro and animal studies and even fewer human studies, reviews, or clinical trials exploring this topic. Additionally, the limited existing literature tends to focus on genetic-based analyses of this protein, rather than its serum concentration and its influence on the serum concentrations of other vitamin D metabolites. Given the increased recognition of the potential role of VDBP in human health, coupled with more accurate means of measuring this protein, and several newly published studies in the context of pregnancy, an updated comprehensive review focused on VDBP in pregnancy is timely.

The purpose of this review is to collate and summarise current evidence regarding the role of VDBP in pregnancy and its influence on pregnancy outcomes, as well as to highlight areas that require further study.

## 2. Overview of Vitamin D

### 2.1. Vitamin D Sources and Metabolism

Vitamin D is a pleiotropic fat-soluble hormone, primarily produced endogenously via dermal synthesis, whereby skin exposure to ultraviolet B rays from sunlight trigger its production [11]. Diet is considered a poor source of vitamin D, with few foods such as fatty fish (e.g., mackerel) and fortified substances (e.g., milk or orange juice) containing vitamin D. Vitamin D can also be derived from the use of ergocalciferol or cholecalciferol supplements, the latter being more common [21,22].

Following ingestion or synthesis in the skin, the lipophilic inactive vitamin D is reversibly bound to VDBP, and to a lesser extent to albumin, within the systemic circulation [23]. It is then transported to the liver where it undergoes enzymatic conversion to 25(OH)D. VDBP possesses a single binding site for vitamin D and all its metabolites to bind. However, this often does not pose a rate-limiting challenge in vitamin D metabolism since, in healthy individuals without renal disease, only 3%–5% of the total circulating binding sites are occupied at a single point in time [11].

After the first hydroxylation in the liver, these metabolites are once again reversibly bound to VDBP and transported to the kidneys for activation [20,24]. VDBP is fundamental for the entry of 25(OH)D into renal tubular cells to be converted to the active form [24]. The entry of the 25(OH)D-VDBP complex into the cell is facilitated by receptor-mediated endocytosis involving two proteins, cubilin and megalin, working in tandem [25]. At this step, 25(OH)D is released within the cell and undergoes enzymatic conversion into either the active form of vitamin D, 1,25-dihydroxyvitamin D (1,25(OH)_2_D_3_) or the inactive form 24, 25-dihydroxyvitamin D [26]. These are subsequently carried by VDBP to their target sites. The active 1,25(OH)_2_D_3_ binds to the nuclear vitamin D receptor (VDR) present in most human cells and tissues (including in the liver, kidney, placenta, endometrium, pituitary, ovaries and pancreatic β-cells) in order to exert its biological functions [27]. This active constituent of vitamin D has 1000 times the affinity to the VDR compared to 25(OH)D [24,28]. This is in conjunction to a comparably limited half-life of 4–6 h compared to a half-life of 3 weeks for 25(OH)D [20].

### 2.2. Epidemiology, Definitions, and Causes of Vitamin D Deficiency

Currently, serum concentrations of total 25(OH)D are widely used to define deficiency [29]. The Institute of Medicine (IOM) classifies vitamin D insufficiency as having a serum 25(OH)D between 50 and 74 nmol/L, [30] whereas deficiency is a 25(OH)D level below 50 nmol/L and is further classified by severity (Table 1) [31].

Vitamin D deficiency is highly prevalent, affecting over 1 billion people worldwide, with an estimated prevalence of 20%–60% in the UK and 10%–40% in the US [33]. Even in countries with a generally sunny climate, including Australia, India and Saudi Arabia, deficiency is prevalent at rates of 30%–50% [1,34,35,36,37]. During pregnancy and lactation, there is an increased risk of vitamin D deficiency due to increased requirement. Global reports suggest that 40%–98% of pregnant women have 25(OH)D levels below 50 nmol/L and 15%–84% have levels below 25 nmol/L [38,39].

Vitamin D deficiency results mainly from reduced exposure to sunlight, particularly in Northern latitudes and in high-risk populations such as the elderly, home- or institution-bound, or dark-skinned or veiled individuals [40]. Nutritional vitamin D deficiency is also common and can be caused by several factors including dietary inadequacy often associated with veganism or impaired absorption from the digestive tract due to malabsorptive diseases (e.g., coeliac disease, pancreatic insufficiency, cystic fibrosis) [13]. Impaired ability of kidneys to convert vitamin D to its active form or increased excretion in conditions such as nephropathy may also lead to deficiency [13,21,41]. Additionally, drugs such as calcium channel blockers, corticosteroids and anticonvulsants augment the catabolism of 25(OH)D and 1,25(OH)_2_D_3_ and can alter the metabolism of vitamin D, contributing to deficiency states [42].

Ethnic variation and genetics also influence an individual’s capacity to metabolise vitamin D and hence vitamin D levels, as well as the functions of vitamin D and its constituents. Ethnicities associated with darker skin have an increased risk of vitamin D deficiency, with lower free, bioavailable and total 25(OH)D levels [43]. This association is consistent even when controlling for the environment and levels of sunlight exposure, demonstrating a potential impact of genetic variation on vitamin D metabolism and concentrations [44]. For instance, pregnant African-American women are reported to have lower total but not free 25(OH)D [45], while others report that African-American women have a significantly lower concentration of serum VDBP correlating with reduced levels of total 25(OH)D, as well as the largest discrepancy in measurements of calculated free and total 25(OH)D [46]. This further suggests that population/ethnicity-specific algorithms for measuring vitamin D levels may be necessary [46].

### 2.3. Controversy Regarding the Measurement of Vitamin D and Its Metabolites to Determine Vitamin D Status

**Figure 1:**
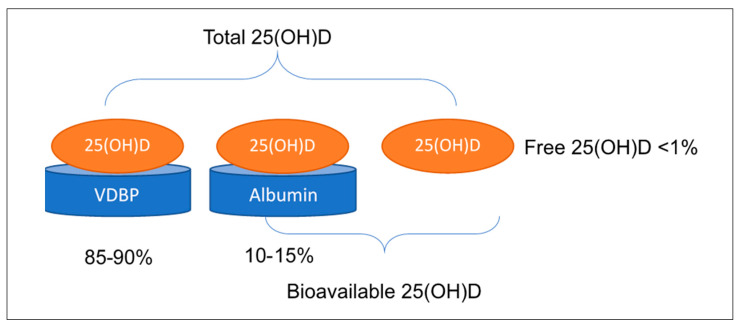
Differentiating between proportions of free, bioavailable and total 25(OH)D in serum: total 25(OH)D reflects 25(OH)D which is bound to vitamin D binding protein (VDBP; approximately 85%–90% of total 25(OH)D) as well as 25(OH)D bound to albumin (10%–15% of total 25(OH)D) and that which circulates free (<1% of total 25(OH)D). Bioavailable vitamin D refers to the sum of free and albumin-bound 25(OH)D (i.e., that which is not bound to VDBP).

Total serum 25(OH)D is the most widely used biochemical marker to determine vitamin D status. However, there is considerable debate regarding whether this is the most appropriate measure to reflect the biological functions of vitamin D and whether free or bioavailable 25(OH)D may be a more accurate measure of status [47,48]. Free or unbound 25(OH)D consists of less than 1% of total serum 25(OH)D, while bioavailable 25(OH)D, which includes a combination of free and albumin-bound 25(OH)D (with much lower binding affinity), makes up approximately 10–15% of total 25(OH)D (Figure 1) [16]. The free hormone hypothesis implies that only the unbound vitamin D is physiologically active and able to exert biological actions, and hence may be a more accurate determinant of functional vitamin D status [47,49]. Indeed, total 25(OH)D concentration often correlates to free 25(OH)D, except in states such as pregnancy where the concentrations of the carrier proteins undergo significant changes themselves [47,48].

Because VDBP levels change drastically during pregnancy, this can influence the concentration of free 25(OH)D as well as other vitamin D metabolites, leading to measurements of total 25(OH)D being no longer representative of vitamin D status in this population [50,51,52]. In fact, this increase in the primary carrier protein is accompanied by an increase in total 25(OH)D and a consistent decline in free 25(OH)D from 15 to 36 weeks gestational age [53]. Thus, despite results showing adequate vitamin D levels or an improvement in vitamin D status throughout pregnancy with supplementation, this may be inaccurate and rather explained by the concomitant increase in total 25(OH)D with the natural increase in VDBP in pregnancy. However, as the free functional portion decreases, the deficient state in pregnant women may be underreported and thus inadequately recognised and treated.

Total 25(OH)D as a single marker is therefore unlikely to be a sufficient means of accurately determining vitamin D status, particularly in physiological states such as pregnancy which involve alterations in metabolism and concentrations of various vitamin D metabolites as well as VDBP. In such states, measuring VDBP and calculating free or bioavailable 25(OH)D may be more valuable [49]. VDBP-bound compounds have limited effect on most target cells and biological activity often correlates with the free hormone concentration, the latter of which may potentially better represent vitamin D status [47,49,54].

VDBP can be measured using various assays, including monoclonal or polyclonal immunoassays and liquid chromatography–tandem mass spectrometry (LC-MS/MS). Recently, the widely used monoclonal enzyme-linked immunosorbent assay (ELISA) was shown to have a potential bias due to technical errors, mainly the effects of genetic polymorphisms and ethnicity, which yield significantly lower VDBP concentrations in Black versus White individuals, unreflective of true genetic differences [55]. In polyclonal immunoassays and LC-MS/MS, less than 9% of the variability in VDBP concentrations measured is attributed to genetic differences, compared to the 85% variability due to genetic differences exhibited in monoclonal immunoassays [55].

Total 25(OH)D may be measured using immunosorbent or chemiluminescent assays as well as LC-MS/MS, the current gold-standard (7, 43). Free 25(OH)D can be calculated indirectly using the concentration of other metabolites, including total 25(OH)D, VDBP and albumin (50). Bikle et al. [47,56,57,58] proposes the following formula for calculating free 25(OH)D:

Free Vitamin D = Total Vitamin D/(1 + ((binding constant albumin) × albumin) + ((binding constant DBP) × DBP))(1)

This calculation was shown to have similar (difference of ~1%) estimates of free 25(OH)D to other proposed formulas [40]. More recently, an immunoassay for the direct measurement of free 25(OH)D has also become commercially available. The assay appears to be better correlated with actual levels of free 25(OH)D than the calculation method in certain ethnicities, such as African American populations [46,57]; however, it requires further validation and standardisation in the general population before it can be adopted into standard clinical practice [59].

### 2.4. Guidelines for Treatment

Oral vitamin D supplementation may be the ideal method for treating deficiency with consideration of public health concerns for safe sun exposure and skin cancer prevention. However, the amount of supplementation needed to obtain extraskeletal health benefits has been widely debated and remains contentious [60,61]. Recommended oral intakes of 200–600 IU daily for adults aged 19–70 conflict with the results of recent studies demonstrating that a minimum oral intake of 4000 IU daily may be required to increase serum 25(OH)D within 2–3 months to optimal levels ≥75 nmol/L [33,62,63]. Furthermore, intakes of 400–600 IU daily in prenatal vitamins, as recommended by the IOM during pregnancy and lactation, have been determined to be inadequate in preventing or treating vitamin D deficiency in pregnancy, especially in women with an existing deficiency and/or limited sun exposure [43,64,65,66].

Current recommendations for optimal 25(OH)D levels to support a healthy pregnancy and the daily supplementation required during pregnancy vary according to the organisation and their respective guidelines (Table 2) [15]. The wide range of recommendations and lack of consensus suggests that perhaps the flaw lies in the methods of measuring and defining vitamin D deficiency and highlights the need for further investigation. This may be significant, particularly in states such as pregnancy where the proportions of the vitamin D metabolites themselves shift considerably. In these states, the single marker of total 25(OH)D may not provide a comprehensive and accurate picture of clinically relevant vitamin D status, highlighting the need to further investigate other components of the vitamin D system in pregnancy.

Continuing supplementation into the postpartum period in breastfeeding mothers is also fundamental due to the high risk of vitamin D deficiency in breastfed infants born to mothers who are deficient during pregnancy [67,68]. The level of vitamin D in breastmilk correlates with maternal vitamin D status, which can then correspond to deficient levels of vitamin D in the neonate, potentially leading to seizures in acute hypocalcaemic states [67,69]. Breastmilk composition also varies dramatically over time, with the colostrum produced in the first 48 h deemed to be the portion most abundant in various proteins including VDBP [70]. Thus, infants who miss this early lactation period may be at increased risk of having a poor vitamin D status and the implications of this, such as a predisposition to neonatal hypocalcaemia in the immediate postpartum period and the development of rickets in the months following, if deficiency is not corrected [71]. Conversely, vitamin D supplementation during pregnancy may positively affect the availability of vitamin D to the fetus and the neonate [72]. 

Although meeting vitamin D requirements is important in pregnancy, vitamin D toxicity may occur when the serum concentration of 25(OH)D exceeds 375 nmol/L [73]. The IOM proposes that an intake of 10,000 IU/day would be required to reach this serum level and cause acute toxicity in adults; however, the serum level at which fetal development and life may be impaired has not yet been determined [74]. Potential risks to the fetus of exogenous vitamin D toxicity by over-supplementation during pregnancy have only been investigated in animal-based studies which linked excess vitamin D intake with adverse outcomes, particularly supravalvular aortic stenosis [75,76,77]. Accurately defining vitamin D status to optimise supplementation but avoid toxicity requires further study to identify how levels of supplementation impact on fetal health and development [73]. 

## 3. Vitamin D Binding Protein

Vitamin D-binding protein, originally known as group-specific component (GC) of serum (GC-globulin), is a protein encoded by the *GC* gene. VDBP is a 58 kDa glycosylated alpha-globulin that is synthesised in the liver, although it can also be expressed in fat tissue, the kidneys, and gonads. It is composed of 458 amino acid residues in length and folds into a triple-domain structure bound by disulphide bonds [20,78]. VDBP is the primary plasma carrier protein to which the metabolites of vitamin D are bound for transport around the body [78]. It is also involved in the chemotaxis of other molecules such as fatty acids and endotoxins, and has immunomodulatory properties. Importantly, VDBP is a component of the actin scavenger system that augments the pro-inflammatory response and clears the products of tissue injury, and it influences T cell responses and the VDBP-macrophage activating factor (DBP-MAF) which is involved in bone metabolism [79]. 

There are many single-nucleotide polymorphisms (SNPs) in the *GC* gene and combinations of two of these *(rs7041* and *rs4588)* result in three polymorphic alleles and the six major phenotypes [80]. These phenotypes differ amongst individuals and in distribution between races [81]. These alleles have also been shown to impact the binding affinity of VDBP to vitamin D metabolites, influencing the proportion of functional free 25(OH)D and deemed to further impact the levels of the carrier protein itself and serum total 25(OH)D as a result [80]. Indeed, specific alleles have been associated with concentrations of VDBP and total 25(OH)D and an increased risk of developing a variety of adverse pregnancy outcomes, including low birthweight of infants and pre-eclampsia [82,83,84].

### VDBP in Pregnancy and Lactation

VDBP is a vital component of the vitamin D system and has been shown to increase drastically during pregnancy, though studies depict this increase to be of varying magnitudes, with some reporting an increase as high as 40%–50% compared with non-pregnant women [53]. At the early third trimester or around 28 weeks gestation, serum VDBP concentrations reach their peak [85] and are almost twice the postpartum level [53]. This increase in VDBP is associated with an increase in total 25(OH)D and a decrease in free and bioavailable 25(OH)D, with the lowest level of free 25(OH)D mapped to occur at approximately 36 weeks gestation [40,53]. This pattern of change, with the capacity to store and metabolise more vitamin D, is thought to enable pregnant women to maintain a sufficient concentration of vitamin D throughout pregnancy and lactation despite the states of increased requirement to support the growing fetus and ensure a healthy successful pregnancy. In vitamin D-deficient women, however, this system is unable to meet the demands of the mother and developing fetus [86].

In pregnancy, the increase in VDBP is thought to occur in response to rising oestrogen. VDBP has been shown to increase when oestrogens are increased, such as in pregnancy, in high stress states, in some ovarian tumours and with hormone replacement therapies [85]. The purported significance of oestrogen was studied by Van Hoof et al. [87] in a population of 38 women who consumed oral oestro-progestogens for three months. They exhibited a significant rise in VDBP and total 25(OH)D, although free 25(OH)D concentrations remained unchanged [87,88]. These findings support the theory that it may be the oestrogen changes during pregnancy that drive the high VDBP state, particularly in the third trimester when oestrogen levels are at their highest [87]. 

The placenta, an organ unique to pregnancy via which the fetus indirectly (originally sourced maternally) obtains their only supply of vitamin D, has also been shown to express VDBP [89,90]. Placental cells express the components necessary for vitamin D signalling including the VDR and VDBP and can synthesise and respond to 1,25(OH)_2_D_3_ and 24,25(OH)_2_D [91]. It is currently unknown whether maternal vitamin D compounds enter placental cells by the endocytosis of 25(OH)D-VDBP, by diffusion of the free hormone, or via both mechanisms. However, the discovery of the expression of megalin and cubilin on the surface of placental cells; fundamental for the receptor-mediated endocytosis of the 25(OH)D-VDBP complex into target cells to be transformed into 1,25(OH)_2_D_3_ or 24,25(OH)_2_D; may highlight the significant influence of VDBP on fetal vitamin D levels [91]. Without VDBP, the maternally derived 25(OH)D may be unable to enter the placental cells to be transformed into the active form of vitamin D and be transported to the fetus for utilisation.

After delivery, mother and neonate undergo a drastic decrease in total 1,25(OH)_2_D_3_ and VDBP, postulated to occur due to the sudden concurrent reduction in oestrogen levels [92]. These titres only rise gradually during the course of lactation to reach equivalent levels to non-pregnant non-lactating women at 18 weeks postpartum [86].

## 4. VDBP and Fertility-Related Outcomes

In recent years, some studies have explored VDBP in relation to fertility-related and pregnancy outcomes, albeit in a limited number of human studies. An overview of these studies is presented in Table 3 and summarized in the following sections.

### 4.1. VDBP in Fertility and Assisted Reproduction

Infertility is defined as the failure to establish a clinical pregnancy following a period of 12 months of unprotected, regular intercourse [93]. It affects 8%–12% of couples of reproductive age worldwide [93]. Infertility in women is associated with conditions such as polycystic ovary syndrome, endometriosis and sexually transmitted infections [93]. From a mechanistic perspective, in vitro experiments have implicated vitamin D in fertility, demonstrating its involvement in regulating embryo-implantation and, to a lesser extent, folliculogenesis [94]. Impaired oocyte maturation, egg development and fertilisation in healthy women, as well as arrested follicular development and menstrual dysfunction may result from abnormal calcium and PTH homeostasis, secondary to vitamin D deficiency [95]. Regulation of calcium by the vitamin D system is also important for sperm motility and acrosome reactions, which are vital for oocyte penetration [96]. Non-calcitropic mechanisms of vitamin D have also been reported. In males, vitamin D reduces triglyceride content and increases lipase activity in sperm to support energetic demands for sperm capacitation [97]. In females, vitamin D plays a role in placental steroidogenesis and the decidualization of the endometrium to support fertility [98]. Hormones including estradiol, progesterone, human chorionic gonadotrophin, and human placental lactogen are also regulated by the vitamin D system, and are important for maternal immunotolerance and regulating utero–placental blood flow and neovascularisation [98]. 

A recent cross-sectional study concluded that vitamin D insufficiency negatively affects fertility and successful clinical pregnancy rates in women undergoing in vitro fertilisation (IVF) [99]. The effect was thought to be mediated at both the ovarian and endometrial level with a lower rate of women reaching the stage of blastocyst transfer and, of those that did, deficient women had lower chances of successful embryo implantation [99]. VDBP itself is proposed to influence fertility due to its known immunological role in maintaining an environment of tolerance to fetal and paternal tissue and their alloantigens. A case-control pilot study of 68 women found that VDBP concentrations were in fact lower in infertile women compared to fertile women [100]. VDBP has also been considered an important biomarker when conducting IVF in women struggling to conceive. Using LC-MS/MS, VDBP was identified as a protein biomarker with the potential to calculate the likelihood of livebirth. Specifically, higher abundance of the VDBP protein was found in the group who successfully achieved live births after the IVF regime compared with the unsuccessful group [101].

The relationship between the vitamin D system and IVF outcomes may, in part, be explained by ethnic variation. Women of South Asian ethnicities have consistently lower vitamin D status and have also been shown to have poorer IVF outcomes compared to Caucasian women [102]. This generated interest as to whether vitamin D status may be a factor involved in the success of IVF [103]. Indeed, previous studies on primarily Caucasian populations demonstrated that the odds of pregnancy were four times higher in vitamin D-replete women compared to -deficient women [104]. However, further study found the inverse evident in East Asian women, whereby elevated total 25(OH)D, particularly an elevated bioavailable fraction, was associated with poorer IVF outcomes [104,105]. This was suggestive of a complex genetically derived relationship between vitamin D and its carrier proteins, particularly VDBP, as it influences the bioavailable fraction of vitamin D [100]. VDBP has not been adequately addressed in the infertility literature, despite increased recognition of its influence on vitamin D metabolite concentrations and that polymorphisms in the VDBP gene (which vary among ethnic groups) result in varied affinity for vitamin D [100]. Further study may help explain the ethnic disparities in IVF outcomes and may be useful for tailoring ethnicity-specific approaches to treating vitamin D deficiency in women with infertility. 

### 4.2. VDBP and Polycystic Ovary Syndrome

Polycystic ovary syndrome (PCOS) is a heterogeneous endocrine disorder with an array of health complications including menstrual dysfunction, hirsutism, metabolic syndrome and infertility/subfertility [135]. PCOS has a reported prevalence varying from 8%-18% worldwide in reproductive-aged women depending on the criteria used, and thus represents a large proportion of the burden of infertility in women [136]. Vitamin D deficiency is proposed to be associated with PCOS, as expertly reviewed elsewhere [137]; however, whether vitamin D deficiency is a cause or consequence of PCOS remains unclear. Vitamin D deficiency may exacerbate PCOS as it has been associated with important features of PCOS such as obesity and insulin resistance [137]. At the molecular level, the vitamin D system may be involved in regulating anti-mullerian hormone (AMH) production and signal transduction, which is important in oocyte maturation and is elevated in women with PCOS [138]. Studies indicate that vitamin D alters granulosa cell differentiation and luteinisation potentiation and decreases receptor expression and sensitivity to follicle-stimulating hormone and AMH while promoting progesterone secretion [138]. Collectively, these mechanisms suggest a role for the vitamin D system in follicle development, menstrual function, and hormone regulation, all of which are key processes underlying the pathology of PCOS.

In relation to VDBP specifically, a recent cross-sectional study of 149 women [106] found that women with PCOS had similar bioavailable and free 25(OH)D levels but lower total 25(OH)D and VDBP concentrations compared with controls, independently of BMI, age, and insulin resistance [106,108]. Negative correlations between VDBP and cardiovascular risk factors, abdominal fat deposit, BMI, and fasting glucose were reported, as well as a positive correlation with HDL cholesterol [106,107]. Furthermore, the obese subgroup had a lower concentration of VDBP compared to controls, whilst the normal weight group with PCOS had comparable levels of VDBP compared to their matched control group [107]. A pilot study of 63 women found no differences in VDBP between women with (*n* = 27) or without PCOS (*n* = 36); however, VDBP was significantly associated with bisphenol A (BPA) in women with PCOS [110]. BPA is a monomer which interacts with estrogen and androgen receptors and has been linked to the pathogenesis of PCOS [110]. The authors posit that the relationship between BPA and VDBP in women with PCOS may be explained by liver dysfunction, as illustrated by the higher bilirubin and aspartate/alanine transaminase (AST/ALT) ratio in women with PCOS compared with controls [110]. In a genetic study, SNPs *rs7041* and *rs2060793* of the *GC* gene in vitamin D-deficient women were associated with an increased risk of developing PCOS in a population of Indian women [108]; however, a case-control study of genotypes and allele frequencies of VDBP polymorphisms (*rs4588, rs7041*, and *rs22822679*) found no differences between women with and without PCOS in a sample of 1359 women [109]. Hence, while there may be a possible association between VDBP and the aetiology of PCOS, further studies are needed to clarify this association and its underlying mechanisms.

Another condition related to infertility is endometriosis. Endometriosis occurs when endometrial tissue (glands and stroma) develops in locations outside of the uterus, causing chronic pelvic pain, dysmenorrhea, dysuria, dyspareunia, dyschesia, and subfertility [139]. With an average annual incidence rate of 7.2 per 10,000 and studies reporting infertility or subfertility in approximately 37% of established cases, endometriosis is a leading cause of infertility [140]. VDBP has been proposed as a potential biomarker for distinguishing women with and without endometriosis, as urinary VDBP levels are elevated in women with endometriosis [111]. Decreased expression of one VDBP isoform (DBPE) was also discovered in the peritoneal fluid of patients with endometriosis compared with patients without the disease [112]. Further inquiry revealed that DBPE levels in the peritoneal fluid of patients with endometriosis treated with oral contraceptive pills (oestrogen and progesterone) were significantly higher than in untreated women [112]. Conversely, serum VDBP and VDBP gene polymorphisms did not differ between women with mild or advanced endometriosis compared with healthy controls in two studies of a single small cohort of 32 women [113,114]. While VDBP may play a role in the pathogenesis of endometriosis and may have potential as a non-invasive biomarker of the disease, its use as a diagnostic tool is limited by a lack of rigorous research in this area, and further investigation is required to identify the role, if any, that VDBP plays in endometriosis [111,141].

### 4.3. VDBP and Pregnancy Loss or Miscarriage

VDBP levels have also been implicated in spontaneous miscarriage [115]. Spontaneous miscarriage is the natural death of the fetus before 20 weeks gestation and occurs in approximately 20% of confirmed pregnancies [115]. VDBP was one of three proteins with increased placental and decidual expression [115]. This was the first report of altered utero–placental vitamin D catabolism in spontaneous miscarriage demonstrating a role for the complex vitamin D metabolic system in the pathology of this debilitating condition.

High VDBP concentrations have also been associated with unexplained recurrent pregnancy loss (URPL). URPL, which is defined as having two or more consecutive pregnancy losses, affects approximately 5% of couples and often has no determinable cause [142]. One study found that VDBP was increased in the placentae of women with URPL compared with normal placentae [116]. The increased expression of VDBP on the surface of placental cytotrophoblasts during pregnancy may be further upregulated in URPL as a mechanism for scavenging the actin filaments resulting from the tissue injury and for the control of excess cytotrophoblast proliferation [116]. VDBP, with its known role in these mechanisms, is therefore thought to be involved in placentation and the pathophysiology of URPL.

Overall, there appears to be a somewhat delicate balance, whereby VDBP levels which are too high or too low may directly or indirectly by its effect on vitamin D levels, be associated with unfavourable consequences in relation to fertility and successfully achieving pregnancy. Further studies are required to identify the limits of this delicate balance and to delineate the potential use of VDBP as a biomarker for fertility outcomes or target for fertility-related therapies [116].

## 5. VDBP and Pregnancy Outcomes

### 5.1. VDBP and Preeclampsia

Pre-eclampsia is a detrimental pregnancy condition defined after 20 weeks gestation by the development of hypertension (blood pressure >140/90 mmHg) and either proteinuria (spot urine protein/creatinine >30 mg/mmol or >300 mg/day) or evidence of other maternal organ dysfunction (creatinine >90 µmol/L, elevated transaminases, altered mental state, severe headache, hyperreflexia, clonus, thrombocytopenia) [143]. It affects 3.0%–3.3% of pregnant women and has many implications for both mother and offspring, often leading to premature delivery [144,145].

SNPs of three genes involved in vitamin D metabolism, including *GC*, have been implicated in pre-eclampsia risk [117]. The *GC*-1 phenotype in particular was identified as a potential early detection genetic marker for women at risk of pre-eclampsia, being more prevalent in women with pre-eclampsia compared to pregnant women without the disorder [117]. In an HIV endemic region of South Africa, and independent of HIV status, two SNPs of the *GC* gene (*rs4588* and *rs7041*) were more frequently present in women whose pregnancies were complicated by pre-eclampsia compared with normotensive pregnancies [121]. In both early-onset (<34 weeks gestation) and late-onset pre-eclampsia (≥34 weeks gestation), *rs4588* was more prevalent, while *rs7041* was more prevalent in early-onset pre-eclampsia compared with normotensive pregnancies [121].

Various studies attempting to better understand the pathophysiology of pre-eclampsia have shown that several proteins, including VDBP, have different plasma concentrations in women who developed pre-eclampsia compared to pregnant normotensive controls [118,146]. A nested case-control study of 170 American women from the Massachusetts General Hospital Obstetric Maternal Study [147] tracked VDBP and total 25(OH)D throughout pregnancy to examine whether these biomarkers were associated with blood pressure or risk of pre-eclampsia. No significant correlation between VDBP or 25(OH)D levels and pre-eclampsia were found [147]. Ma et al. [89] performed a study of 34 placentas, 17 from normal-term pregnancies, 11 from pre-eclampsia, three from first-trimester pregnancies and three from second-trimester pregnancies [89]. In this study, in vitro cultures of primary isolated trophoblasts were put under increased oxidative stress akin to the underlying pathophysiology of pre-eclampsia [89]. They found that the increased oxidative stress correlated with a downregulated expression of certain proteins including VDBP, suggesting that increased oxidative stress may be responsible for the altered concentration of VDBP and vitamin D metabolism evidenced in the placentae of women with pre-eclampsia [89]. Moreover, pregnancies complicated by pre-eclampsia with proteinuria have been shown to result in a greater urinary loss of VDBP compared to non-proteinuric pre-eclampsia or normotensive pregnancies, suggesting a possible disruption to the vitamin D metabolism and function through reduced VDBP [123].

The pathophysiology of pre-eclampsia is believed to have an inflammatory, immunological and potentially auto-immune component. A role for VDBP in the auto-immune component of pre-eclampsia was suggested when VDBP of placental origin was identified as an autoimmune target of auto-antibodies in the sera of 20 women with pre-eclampsia compared with the sera of 20 healthy non-pregnant women [119]. Another way in which VDBP may be implicated in pre-eclampsia is via its actin scavenging role. Plasma gelsolin and actin free VDBP (AFVDBP), established components of the actin clearance system, were investigated in a longitudinal study of 40 women [120]. Women with a normal pregnancy exhibited a significant reduction in plasma gelsolin and a concomitant rise in AFVDBP; however, pregnant women with pre-eclampsia (four early onset pre-eclampsia defined as <33 weeks gestation and 11 late onset pre-eclampsia at ≥35 weeks gestation) did not exhibit the expected fall in serum gelsolin and rise in AFVDBP [120]. This suggests that pre-eclampsia is a state of dysfunctional and depleted actin clearance in which VDBP may be implicated [120].

Moreover, Emerson et al. [122] in a study of 126 pregnancies, 100 normal and 26 with pre-eclampsia, discovered that GC:actin complexes were present in larger concentrations in women with complicated pregnancies compared with healthy pregnancies. These experiments showed increased actin release from trophoblast membranes and lysis of other cells in physiological states such as pre-eclampsia [122]. The reliability of these findings is limited by the absence of a quantitative method for measuring GC:actin, since the isoelectric focusing method used in the study is acknowledged to be less accurate, potentially underestimating the degree of complex formation [122]. Collectively, these studies suggest that VDBP may be involved in the pathogenesis of pre-eclampsia via its role in the actin clearing system and the scavenging of actin released by damaged cells [122]. The direction of the association, whether the alteration in the system is responsible for inciting pre-eclampsia or if pre-eclampsia changes the VDBP-mediated actin-clearing system, however, requires further study.

### 5.2. VDBP and Gestational Diabetes Mellitus

Gestational diabetes mellitus (GDM), defined as glucose intolerance first developed or recognised in pregnancy, affects 10% of pregnancies in Australia and up to 30% of pregnancies in high-risk populations [148]. GDM is diagnosed using oral glucose tolerance tests, generally at 26–28 weeks gestation; however, timing, diagnostic criteria and target individuals for screening differ by country and guidelines. The development of GDM has been associated with the vitamin D system, potentially involving several variant alleles of vitamin D signalling pathways including alleles of VDBP [149,150]. In a case-control study of 964 GDM cases and 1021 controls, the *GC rs16847024* allele was found to be significantly associated with an increased risk of GDM, whereby the risk of developing GDM increased in a significant dose-dependent manner with the number of variant alleles present [151]. Similarly, *GC rs3733359* was also associated with an increased risk of GDM in obese pregnant women in an analysis of 1494 pregnant women [124]. Notably, both studies only included women of Chinese ethnicity, warranting further validation studies in multi-ethnic populations [151].

The only study investigating a potential relationship between serum VDBP concentration and the development of GDM is a recent nested case-control study by Xia et al. [19]. This study included 107 cases of GDM and 214 pregnant controls and reported no association between VDBP and GDM; however, this may have been due to some limitations of the study, including the use of monoclonal assay technology to measure VDBP [19].

Although very few studies have been conducted examining VDBP in GDM, links between the vitamin D system and metabolic health have been supported by animal and human studies in obesity and type 2 diabetes mellitus (T2DM). While pregnancy itself may not influence the bioavailability of vitamin D [152], obese pregnant women are more likely than non-obese women to have inadequate levels of total 25(OH)D in pregnancy [153]. This may be due to sedentary lifestyles and lack of sun exposure, or could result from the sequestration of vitamin D in body fat or from volumetric dilution (greater volume of distribution in tissue mass) [5]. Vitamin D also promotes PTH and calcium flux into adipocytes, which facilitates lipogenesis, potentially promoting weight gain [5]. VDBP has also been associated with fat and carbohydrate metabolism as well as adiposity/obesity [154]. In a study of 43 obese and 43 normal weight women, obese women of reproductive age had higher concentrations of VDBP and lower concentrations, often suboptimal, of the biologically active fraction of vitamin D, free 25(OH)D, compared with their normal weight or underweight counterparts [154]. The exact mechanisms and causal relationships between the vitamin D system and obesity, including in pregnancy, are not fully understood. However, most of the currently available data, including from gene association studies, suggest that obesity leads to vitamin D deficiency, while the reverse relationship is generally not supported.

In relation to diabetes, studies have reported a significant decline in maternal and neonatal 1,25(OH)_2_D_3_ and VDBP after induction of diabetes in rats, in support of the theory that insulin deficiency influences the vitamin D system [155,156]. Furthermore, in androgen- or oestrogen-deficient diabetic rats, it was the treatment of insulin deficiency rather than of androgen or oestrogen deficiency that resulted in the restoration of VDBP and 1,25(OH)_2_D_3_ levels, although sex hormones mediated some of the changes in VDBP and vitamin D metabolites [155]. These findings suggest a complex relationship between sex steroids and insulin, with insulin deficiency having deleterious effects on vitamin D metabolism, facilitated by androgens and counteracted by oestrogens. Conversely, a human-based cross-sectional study by Wang et al. [157] demonstrated an inverse relationship between VDBP and insulin levels in 47 obese post-menarchal female adolescents, and a positive relationship between VDBP levels and whole-body insulin sensitivity. This association persisted after correction for adiposity and race, suggesting a direct but multifactorial relationship between insulin resistance and VDBP in humans.

Wang et al. [158], in a meta-analysis including six studies with 1191 T2DM cases and 882 healthy controls, further identified that SNPs *rs7041* and *rs4588* were both associated with an increased risk of T2DM, however, only in Asian populations [158]. Several limitations were noted pertaining to the inability to control for gene–gene and gene–environment interactions, despite the recognised influence of multiple genetic and environmental factors in the development of this complex metabolic disorder [158]. With no apparent relation between VDBP phenotypes and the development of T2DM in Caucasians, but evidence of an association in Asian populations, this poses an intriguing area for further research to examine ethnic variation and determine its significance in clinical practice, particularly in the context of pregnancy and GDM.

### 5.3. VDBP and Preterm Birth

Preterm birth, which is commonly defined as birth at less than 37 weeks gestation, is a prevalent pregnancy complication, affecting approximately 11% of births worldwide [159]. Preterm birth complications are the leading cause of death among children less than five years of age [159]. Premature/prelabour rupture of membranes (PROM), which is the rupture of the membranous sac encasing the fetus before the onset of labour, occurs in 30% of these preterm births [160]. When PROM occurs before 37 weeks of completed gestation, it is called preterm premature rupture of membranes (pPROM) and is the leading identifiable cause of premature births [160]. Hence, the ability to detect and quantify the risk of preterm labour, and PROM as a leading cause, has been an important focus of study. Increased VDBP in cervicovaginal fluid (CVF) has recently been identified as a potentially relevant biomarker for identifying and understanding preterm births.

A single-centre retrospective cohort study [126] examined VDBP in samples of CVF immediately following amniocentesis collected from women after preterm labour. The study, involving 148 women with preterm labour and 103 with pPROM between 23–34 weeks gestation, found that an increase in concentration of VDBP in CVF independently predicted imminent preterm delivery in women with preterm labour [126]. Pereira et al. [128] also examined 205 proteins in the CVF of 18 women in search of novel non-invasive biomarkers of preterm labour and identified VDBP as being differentially expressed in women who experienced spontaneous preterm birth compared with controls [128]. This was further supported by Liong et al. [18] who discovered that two isoforms of VDBP were up-regulated by over three-fold (ranging from 3.7–3.9-fold) in the CVF of women with spontaneous preterm birth compared with a control group of asymptomatic pregnant women [18]. In agreement with these findings, a longitudinal observational study by the same group [130] investigated 221 healthy pregnant women who spontaneously delivered either preterm or at term. This study demonstrated a 4.3-fold rise in CVF VDBP up to three days prior to premature labour, compared with a 3.4-fold rise in VDBP levels at 15–28 days before labour [130]. It should be noted that a significant proportion of twin gestations were included in the preterm group, which may have influenced these results. In fact, when the singleton and twin groups of both term and preterm groups were subsequently compared, there was no significant difference in the concentration of VDBP [130].

In a study of 15 asymptomatic women who later went on to develop pPROM, VBDP was one of two proteins in the CVF to be significantly elevated in this impending pregnancy state [18]. VDBP and other proteins identified in this analysis are responsible for similar functions including oxidative balance, anti-inflammatory activity, protease inhibition, and metabolism, all of which are mechanisms underlying membrane rupture [18]. A study conducted in Australia used 2D electrophoresis techniques to analyse the CVF proteome and showed that a dual biomarker model of albumin/VDBP is more efficacious than fetal fibronectin (fFN), the most commonly employed gold-standard biomarker for predicting spontaneous preterm delivery in symptomatic women within seven days [17]. Another recent study [127] published in 2020 investigated serum VDBP as an early biomarker for preterm birth. Here, D’Silva et al. [127] examined four different proteoforms which appeared to be strong candidate biomarkers for preterm labour in pregnant women, and found that only one of them, VDBP, was reduced in women who delivered preterm compared to women who delivered at term [127].

## 6. VDBP and Offspring Outcomes

### 6.1. VDBP and Neonatal Vitamin D Status

Maternal VDBP has been implicated in fetal concentrations of VDBP and, in turn, neonatal and fetal outcomes. The significance of VDBP on fetal vitamin D status and during infancy was shown in an observational study by Bouillon et al. [131] comparing levels of VDBP and total 25(OH)D concentrations in maternal serum and cord blood of 30 healthy Belgian mothers and their infants born at term (range 38–42 weeks). Maternal serum had a significantly higher concentration of VDBP and total 25(OH)D compared to the fetus, demonstrating a maternal–fetal gradient for transport of 25(OH)D to the fetus [131]. However, fetal cord blood had a slightly higher concentration of free 25(OH)D compared to maternal serum, suggesting that non-optimal transfer of the carrier protein, rather than the transfer of vitamin D itself, is responsible for neonatal deficiency in normal pregnancies [131]. This highlights that, due to the reduced VDBP and vitamin D stores of infants, optimising maternal vitamin D status during pregnancy through accurate measurement, classification, and supplementation is necessary to support fetal development and prevent adverse neonatal outcomes [131].

### 6.2. VDBP and Fetal Growth and Birthweight

One such outcome is fetal growth restriction (FGR). FGR occurs when the fetus does not reach its biological growth potential, usually due to impaired placental function, which may occur as a result of a myriad of factors including ethnicity, maternal age and concurrent pathologies [161]. FGR affects 3%–7% of newborns and increases the risk of perinatal morbidity and mortality [162], as well as contributing to both short- and long-term difficulties in terms of feeding, respiration and metabolism [163]. Recently, VDBP has been implicated in FGR, albeit with limited evidence [132]. A single retrospective study conducted by Wookey et al. [132] found that VDBP concentrations from 17 placentae of healthy control pregnancies were significantly higher than in 18 placentae from gestation-matched pregnancies complicated by FGR [132]. The direction and extent of this potential relationship is not yet known and, given that there is only one small study supporting a link between VDBP and FGR, this area requires further investigation. Future studies should assess if VDBP can be utilised as a biomarker for FGR before the fetus begins to exhibit signs of FGR, as this would provide a valuable tool to expedite management and take further precautions to prevent detriment to the fetus.

Another neonatal outcome VDBP has been linked to is birthweight. Variant *GC* alleles *rs12512631* and *rs7041* were proposed to indirectly influence infant birthweight in a genotyping study involving 356 pregnant women and their infants [84]. The alleles *rs12512631* and *rs7041* were associated with reduced maternal and cord blood 25(OH)D measured using LC-MS/MS and were also significantly associated with decreased birthweight [84]. Although suggestive of a relationship between VDBP and/or 25(OH)D with birthweight, replication is needed to confirm these findings and explore their wider impact.

### 6.3. VDBP and Infant Health

VDBP in pregnancy has also been linked to the development of type 1 diabetes mellitus (T1DM) in the offspring, a disease in which the pancreas does not produce insulin, resulting in a state of hyperglycaemia [164]. Sorensen et al. [129] conducted a nested case-control study with 113 mother–child pairs recruited from the Norwegian childhood diabetes registry, and found that there was an association between low maternal concentrations of VDBP, particularly in the third trimester, and T1DM in the offspring. This finding was supported by another cohort study where 189 mother/child pairs with offspring who later developed T1DM were compared with 576 random control mother/child pairs [134]. The study found that higher maternal VDBP levels measured from the cord blood at the time of delivery were associated with a reduced risk of T1DM in offspring [134]. This relationship is supported by studies in non-pregnant populations [165], which report higher serum VDBP levels in healthy individuals compared with patients with T1DM or their first degree relatives, as well as links between the development of T1DM and the VDBP genetic variant *rs7041* [166,167]. These studies suggest that components of the vitamin D metabolic pathway may be altered in the development of T1DM and could have the potential to influence disease pathogenesis, potentially during the pregnancy period.

Another pathology which has been linked to VBDP, particularly a specific allelic variant of it, is autism spectrum disorder (ASD). ASD represents a range of conditions in which individuals have persistent deficits in social communication and social interactions in multiple contexts [168]. The aetiology of ASD is still largely unknown [168]. Previously, vitamin D deficiency during pregnancy was linked to an increased risk of ASD [169,170,171,172]; however, it was only recently in a study by Schmidt et al. [133] that the *GC* variant allele *rs4588* in children was associated with the development of ASD [83]. This potential connection requires further large-scale, population-based studies to confirm whether VDBP and its related genetic components may clarify some of the aetiological components behind this disease.

## 7. Critical Appraisal: Limitations and Future Directions

Although vitamin D has been studied extensively in the context of pregnancy, current knowledge regarding the role of VDBP in pregnancy remains sparse. As summarised in Table 2, some studies have implicated VDBP in the pathophysiology of common pregnancy complications and adverse outcomes, either directly or indirectly via its influence on the concentration of free and bioavailable vitamin D. However, this area of research remains unexplored and the relationship between VDBP and pregnancy outcomes is largely unknown.

The exploration of VDBP in pregnancy has gained some traction in recent years, yet, published studies and their findings remain limited by several factors. Most initial studies were animal-based, utilising rodent models and, while these studies present intriguing findings, results are difficult to extrapolate or generalise to human populations. Of the existing human studies, most are limited by small sample sizes, minimal ethnic variation, and lack of adjustment for potential confounding variables (including BMI, age, co-morbidities, ethnicity, and lifestyle factors (e.g., smoking)), all of which can affect both VDBP levels and pregnancy outcomes. Ethnicity in particular is known to be associated with genetic polymorphisms in VDBP and its encoding genes (*GC*), which can influence not only VDBP concentrations, but also its binding capacity and function. Despite this, a paucity of human studies have incorporated ethnic data into their analyses.

Furthermore, recent studies have investigated the measurement methods and technologies commonly employed to evaluate the associations between pregnancy outcomes and VDBP and found them to be flawed [81,173]. Most existing studies on VDBP have used monoclonal immunoassays to measure the level of VDBP. However, these assays have different selective affinity for VDBP genotypes (which vary by ethnicity), and hence the reported findings may have been skewed. Polyclonal assays are proposed to be superior in measuring VDBP and subsequently calculating free vitamin D, and should therefore be utilised in future studies, particularly in studies of multi-ethnic populations [174,175,176]. Many studies investigating VDBP and pregnancy outcomes have also primarily focused on a direct link between VDBP and pregnancy complications, and most did not consider an alternative indirect link. Indeed, the free hormone hypothesis suggests that only free vitamin D (which is highly dependent on VDBP levels and binding affinities) exerts biological functions, but free vitamin D has scarcely been assessed in pregnancy.

## 8. Conclusions

In summary, VDBP has been implicated in pregnancy and reproductive health, but its exact role is not yet fully understood. Study limitations including small sample sizes, inadequate measurement methods and lack of control for confounding factors preclude our ability to draw firm conclusions from the current evidence. More focused studies are needed to address these limitations in order to disentangle the functions of VDBP and to clarify its role as a measure of vitamin D status and an important novel biomarker of pregnancy and reproductive outcomes.

## Figures and Tables

**Table 1:** Definitions of vitamin D deficiency according to the Australian and New Zealand Bone and Mineral Society and Osteoporosis Australia [32].

Status	Definition
Vitamin D adequacy	≥ 50 nmol/L *
Mild vitamin D deficiency	30–49 nmol/L
Moderate vitamin D deficiency	12.5–29 nmol/L
Severe vitamin D deficiency	<12.5 nmol/L

* These levels should be 10–20 nmol/L higher at the end of summer as a buffer to allow for seasonal decrease.

**Table 2:** Recommendations for total 25(OH)D concentrations and daily supplementation doses during pregnancy by international health organisations, 2016 (derived from Mousa, et al. [15]).

Organisation (Country)	Recommended 25(OH)D Level (nmol/L)	Daily Recommended Supplementation Dose (IU)
World Health Organization	>50	200
Institute of Medicine (US)	≥30	600–1000
Endocrine Society (US)	≥75	1500–2000
ACOG (US)	≥50	600
NICE (UK)	>30	400–800
National Institutes of Health (US)	>50	600
RANZCOG (Australia/NZ)	>50	400–2000

Abbreviations: ACOG, American College of Obstetricians and Gynaecologists; NICE, National Institute for Health and Care Excellence; RANZCOG, Royal Australia New Zealand College of Obstetricians and Gynaecologists.

**Table 3:** Summary of studies examining vitamin D-binding protein in relation to fertility and pregnancy-related outcomes.

Condition	Author, Year [Reference]	Participants	Key Findings	Implications	Limitations
Infertility	Franasiak, 2017 [100]	*N* = 68 women; 39 infertile premenopausal, 29 fertile premenopausal controls	Lower VDBP in the infertile group compared to fertile group	VDBP as a potential biomarker for screening/ diagnosis of infertility	Pilot study; small sample size; limited ethnic diversity
In vitro fertilisation (IVF)	Estes, 2009 [101]	*N* = 20 women; 10 with successful IVF (livebirth), 10 without a successful IVF (no pregnancy)	VDBP reduced in unsuccessful IVF candidates	VDBP as a biomarker for good versus bad responders in IVF	Small sample size, single-centre, use of 2D-PAGE methodology to measure proteins considered less accurate than other methods
Polycystic ovary syndrome (PCOS)	Naderpoor, 2018 [106]	*N* = 149 pre-menopausal women; 90 with PCOS, 59 controls	Lower VDBP in women with PCOS than controls. Similar total, free and bioavailable 25(OH)D levels	VDBP as a potential mechanistic biomarker for the development of PCOS	Small retrospective study; VDBP gene polymorphisms were not studied; androgen levels were not investigated; smaller control group
	Kuliczkowska-Plaksej, 2019 [107]	*N* = 267 women aged 20-35 years; 167 with PCOS, 100 controls	Lower serum VDBP levels in obese women with PCOS	Involvement of VDBP in the clinical and biochemical picture of PCOS	Lack of ethnic variation, small sample size
	Haldar, 2018 [108]	*N* = 100 women; 50 diagnosed with PCOS, 50 controls	*GC* alleles *rs7041* and *rs2060793* in vitamin D-deficient women increase the risk of PCOS	Better understanding of PCOS, potential use of VDBP genotypes as biomarkers of PCOS	Small sample size, single ethnicity study, lack of investigation into other enzymes and proteins in the vitamin D system
	Song, 2019 [109]	*N* = 1359 women; 432 women with PCOS; 927 controls	Distribution of genotypes and allele frequencies of the VDBP *rs4588, rs7041*, and *rs22822679* polymorphisms did not differ between PCOS and controls	VDBP polymorphisms do not differ between women with and without PCOS, but further studies are required.	Study power less than 80%; may have missed some metabolic differences between groups; did not account for sun exposure or diet; did not measure circulating vitamin D
	Jedrzejuk, 2019 [110]	*N* = 63 women; 27 women with PCOS; 36 controls	No differences in VDBP between women with or without PCOS but VDBP was associated with BPA only in women with PCOS	Relationship between VDBP and BPA may reflect liver dysfunction in women with PCOS	Small sample size, lack of gold standard methods for measuring vitamin D and VDBP
Endometriosis	Lee, 2011 [111]	*N* = 95 reproductive age women; 57 with endometriosis, 38 controls	Urinary VDBP was elevated in women with endometriosis	VDBP may be involved in the pathophysiology of endometriosis and be a valuable biomarker in detecting the disease alone or in combination with CA-125	Majority of patients in the control group had various other benign diseases which may impact urinary VDBP levels, use of 2-DE methodology
	Ferrero, 2005 [112]	*N* = 145 reproductive age women; 36 untreated mild endometriosis, 52 untreated severe endometriosis, 17 endometriosis treated with oral contraceptives, 40 controls	Reduced expression of one VDBP isoform in peritoneal fluid of women with endometriosis, but improved in women undergoing hormone treatment	VDBP as a biomarker for endometriosis and monitoring treatment of the disease	Small sample size, only patients with mild disease analysed, use of 2-DE methodology with low throughput
	Cho, 2019 [113]; and Baek, 2019 [114]	*N* = 32 women; 9 mild endometriosis; 7 advanced endometriosis; 16 healthy controls (both studies using the same sample, different groupings)	No differences in serum VDBP or in VDBP gene polymorphisms between controls and women with mild or advanced endometriosis	VDBP was not associated with severity of endometriosis; however, further studies are needed	Very small sample size; no assessment of some confounders including sun exposure
Spontaneous miscarriage	Hou, 2020 [115]	*N* = 42 placentas; 20 from spontaneous miscarriages, 22 from normal pregnancies	VDBP was less expressed in the placenta and decidua in spontaneous miscarriages	VDBP as a potential biomarker for miscarriages and its implications in the pathophysiology of spontaneous miscarriage	Small sample size
Unexplained recurrent pregnancy loss (URPL)	Gharesi-Fard, 2014 [116]	*N* = 10 human placentas; 5 URPL, 5 gestation matched controls	VDBP had increased expression in URPL cases	Understanding into the pathophysiology of URPL and the potential use of VDBP as a biomarker	Very small sample size
Pre-eclampsia	Mekbeb, 1990 [117]	*N* = 239 pregnant women; 107 with pre-eclampsia, 132 controls	Increased expression of *GC 2-1* phenotype in women with pre-eclampsia	*GC 2-1* phenotype may be an early detection genetic biomarker for placental dysfunction	Modest sample size; outdated technology that has since been advanced with more accurate methods
	Kolialexi, 2017 [118]	*N* = 10 pregnant women; 5 with pre-eclampsia, 5 controls	VDBP was upregulated in women with early-onset pre-eclampsia in the first trimester	VDBP as a biomarker for screening for early-onset pre-eclampsia	Lack of accuracy with 2D electrophoresis technique; very small sample size
	Ma, 2012 [89]	*N* = 22 human placentas; 11 from pre-eclamptic pregnancies, 11 from normal pregnancies	Increased oxidative stress as occurs in pre-eclampsia resulted in decreased expression of VDBP	VDBP as a biomarker of states of increased oxidative stress such as in pre-eclampsia	Small sample size; lack of correlation between immunostaining and Western blot results in snap-frozen tissues
	Behrouz, 2013 [119]	*N* = 40 women; 5 placentas, 20 sera from normotensive pregnancies, 20 sera from women with severe pre-eclampsia	VDBP of placental origin may be an autoimmune target in pre-eclampsia	Antibodies against VDBP may be involved in the pathophysiology of pre-eclampsia	Use of a 2D-PAGE technique lacks accuracy due to narrow dynamic range and low throughput; small sample size; small sample size
	Tannetta, 2014 [120]	*N* = 40 women; 10 non-pregnant, 10 normotensive pregnancies, 10 early onset pre-eclampsia, 10 late onset pre-eclampsia	Actin free VDBP was dysregulated in pre-eclampsia and lower in early onset pre-eclampsia than in normal pregnancies	VDBP as a biomarker of the underlying mechanisms of pre-eclampsia	Small, single-centre study; lack of statistical power
	Naidoo, 2019 [121]	*N* = 600 pregnant women; 246 normotensive, 167 early onset and 246 late onset pre-eclampsia	SNPs *rs4588* and *rs7041* were present more frequently in pre-eclamptic pregnancies	Genetic biomarkers for pre-eclampsia risk	HIV prevalent region; lack of ethnic diversity
	Emerson, 1983 [122]	*N* = 126 pregnant women; 26 with pre-eclampsia, 100 controls	Increased expression of GC:actin complexes in sera of complicated pregnancies compared with normal pregnancies	Reveals role of VDBP in pre-eclampsia and potential use of GC:actin complexes as biomarkers of complicated pregnancies	Small sample size; source of the actin in the GC:actin complexes in pregnant women could not be equivocally established; needs replication to confirm findings
	Albejante, 2020 [123]	*N* = 20 pregnant women; 15 with non-proteinuric pre-eclampsia; 5 with pre-eclampsia with proteinuria; 10 normotensive controls	Significant loss of VDBP in urine of women with pre-eclampsia with proteinuria	Proteinuria and resultant urinary loss of VDBP in preeclamptic pregnancies may promote vitamin D deficiency	Very small sample size and low statistical power
Gestational diabetes mellitus (GDM)	Wang, 2015 [124]	*N* = 1985 pregnant women; 964 GDM cases, 1021 controls	*GC rs16847024* and *GC rs3733359* were associated with an increased GDM risk	*GC* alleles as potential early genetic biomarkers of GDM risk	Single ethnic group; 25(OH)D not measured in all participants; statistical power was insufficient to detect a small effect size
	Karras, 2018 [125]	*N* = 70 pairs of neonates and their mothers	Maternal VDBP was strongly correlated with maternal adiponectin and neonatal VDBP and adiponectin	Potential independent interaction between VDBP and adiponectin in mothers and neonates. VDBP may be a marker of metabolic health	Small sample size, no association with birth weight
	Xia, 2019 [19]	*N* = 321; 107 GDM cases; 214 controls	Maternal VDBP was not associated with GDM risk at any gestational period (trimester)	No evidence to support the use of VDBP as a biomarker of GDM risk	Use of monoclonal assays to measure VDBP, potential confounding by sun exposure, diet, etc.
Preterm birth	Kook, 2018 [126]	*N* = 251 pregnant women; 148 spontaneous preterm labour, 103 premature prelabour rupture of membranes	Increased CVF VDBP predicted imminent spontaneous preterm delivery within 48 h and intra-amniotic infection in women with preterm deliveries	VDBP may be a biomarker for intra-amniotic infection or impending preterm labour. CVF VDBP may regulate host response to intra-amniotic infection	Lacked comparative data to other predictive tests for preterm labour; molecular technique not used to detect microbes; samples not randomly analysed; confounders not assessed (e.g., recent intercourse, bacterial vaginosis)
	D’Silva, 2020 [127]	*N* = 88 pregnancies; 44 pregnancies that delivered <37 weeks gestation, 44 pregnancies that delivered >37 weeks	Serum VDBP was significantly reduced in the preterm deliveries compared to the term deliveries	Serum VDBP as biomarker for preterm labour and delivery	2DE technique, differences in cohort compared to initial study: delivered 3 weeks later, more ethnic diversity
	Pereira, 2007 [128,129]	*N* = 18 pregnant women; 6 preterm labour without preterm delivery, 6 spontaneous preterm birth without infection, 6 controls	VDBP was upregulated in the CVF of women with spontaneous preterm birth compared with controls	VDBP may be a novel biomarker for preterm birth and improved understanding of the pathophysiology involved in preterm labour and delivery	Small sample size; results may be due to genetic/ biological variation which was not accounted for
	Liong, 2013 [130]	*N* = 221 pregnant women; 48 preterm births, 173 normal term births	Increased expression of VDBP up to 3 days prior to premature labour compared to 15–28 days prior. Increased CVF VDBP in impending preterm and term labour. Unlike fetal fibronectin, CVF VDBP levels were not altered by recent sexual intercourse	VDBP levels may be a more accurate and specific biomarker for predicting labour compared with the gold-standard fetal fibronectin	Small sample size; included several multifetal gestations and did not consider the effects of this on VDBP concentrations
	Liong, 2013 [18]	*N* = 15 pregnant women; 5 with PROM, 10 controls	VDBP significantly increased in the women with PROM	VDBP as a potential biomarker for impending PROM	Small sample size; early and late PROM were combined despite likely different pathophysiologies; confounded by inclusion of both singleton and multifetal gestations
	Liong, 2015 [17]	*N* = 12 women with preterm deliveries; independent cohort of 129 women for ELISA validation	Albumin/VDBP ratio was more efficacious than fetal fibronectin in predicting spontaneous preterm labour in symptomatic women within 7 days	VDBP alone or in combination with albumin as a biomarker to predict preterm labour	Small sample size; confounders such as multifetal gestation, recent bleeding or intercourse were excluded but their impact on CVF expression of albumin and VDBP not determined; women with positive fetal fibronectin received tocolytic therapy which may have influenced results
Hypo-vitaminosis D	Bouillon, 1977 [131]	*N* = 30 maternal–fetal pairs	Fetal cord blood had lower total 25(OH)D and VDBP but higher free vitamin D than maternal blood	Impaired transport of VDBP may result in neonatal vitamin D deficiency; low VDBP intrauterine state is not favourable for the storage of vitamin D in the fetus	Small sample size; outdated technology that has since been improved with more accurate methods
Fetal growth restriction (FGR)	Wookey, 2017 [132]	*N* = 35 human placentas; 18 from pregnancies complicated by FGR, 17 from normal pregnancies	Significantly lower placental VDBP in pregnancies complicated with FGR	VDBP as a potential biomarker for placental dysfunction and FGR	Small sample size; samples only obtained after delivery for analysis, well after peak expression of vitamin D and establishment of placental function; no patient-matched serum samples were used
Reduced birthweight	Chun, 2017 [84]	*N* = 356 paired pregnant women and their neonates	Reduced vitamin D was associated with low birthweight in carriers of *GC rs12512631* allele	*GC* allelic variants as potential genetic predictive biomarkers for low birthweight	VDBP level not calculated; unclear mechanism by which certain *GC* variants modify the relationship between vitamin D and birthweight
Autism spectrum disorder	Schmidt, 2015 [133]	*N* = 1581 children and their parents; 341 children with autism, 1240 controls	*rs4588* was associated with the development of Autism spectrum disorder	Potential use of *GC* allelic variants as risk or even diagnostic markers for autism	Some missing data on paternal genotypes; paternal vitamin D status and levels were not evaluated
Type 1 diabetes mellitus (T1DM)	Sorensen, 2016 [129]	*N* = 333 pregnant women; 113 whose offspring later developed T1DM, 220 controls	Low maternal VDBP in the third trimester was associated with an increased risk of T1DM in the offspring	VDBP as a potential biomarker for T1DM risk	Confounders such as ethnicity not considered; some samples underwent freeze-thaw cycles which may have altered sample integrity
	Tapia, 2019 [134]	N= 767; 189 mother/child pairs where the child later developed T1DM, 576 random control mother/child pairs	Low maternal VDBP levels at birth were associated with an increased risk of T1DM in offspring	VDBP as a biomarker for metabolic risk in offspring such as T1DM	Observational study, possible presence of unknown confounding factors, low external validity due to primarily Caucasian population

Abbreviations: 25(OH)D, 25-hydroxyvitamin D; BPA, bisphenol A; CA-125, cancer antigen-125; CVF, cervicovaginal fluid; ELISA, enzyme linked immunosorbent assay; FGR, fetal growth restriction; GC, group specific component; GDM, gestational diabetes mellitus; IVF, in vitro fertilisation; PCOS, polycystic ovary syndrome; PROM, premature rupture of membranes; T1DM, type 1 diabetes mellitus; URPL, unexplained recurrent pregnancy loss; VDBP, vitamin D binding protein.
